# New Perspectives in Liver Transplantation: From Regeneration to Bioengineering

**DOI:** 10.3390/bioengineering6030081

**Published:** 2019-09-11

**Authors:** Debora Bizzaro, Francesco Paolo Russo, Patrizia Burra

**Affiliations:** Department of Surgery, Oncology and Gastroenterology, Gastroenterology/Multivisceral Transplant Section, University/Hospital Padua, 35128 Padua, Italy; debora.bizzaro@gmail.com (D.B.); francescopaolo.russo@unipd.it (F.P.R.)

**Keywords:** liver regeneration, end-stage liver diseases, regenerative medicine, liver tissue bioengineering, liver bioreactors

## Abstract

Advanced liver diseases have very high morbidity and mortality due to associated complications, and liver transplantation represents the only current therapeutic option. However, due to worldwide donor shortages, new alternative approaches are mandatory for such patients. Regenerative medicine could be the more appropriate answer to this need. Advances in knowledge of physiology of liver regeneration, stem cells, and 3D scaffolds for tissue engineering have accelerated the race towards efficient therapies for liver failure. In this review, we propose an update on liver regeneration, cell-based regenerative medicine and bioengineering alternatives to liver transplantation.

## 1. Introduction

Acute and chronic liver diseases are leading causes of morbidity and mortality worldwide, accounting for about 1–2 million deaths annually [1]. The most prominent causes of acute liver failure include viral hepatitis, alcoholic liver disease, non-alcoholic fatty liver disease (NAFLD), drug-induced liver injury, and autoimmune liver disease [2,3]. 

Liver transplantation is the ultimate solution in the treatment of such severe liver dysfunctions. Despite the relatively high postoperative survival rate, there are many problems to be solved, however, including a chronic donor shortage, immune rejection, and ethical issues. Therefore, cell-based regenerative therapies and novel technologies such as liver-on-chip [4] and bioprinted liver [5] are expected to be the next-generation therapies.

These innovative approaches are all based on the extraordinary capacity of the liver to regenerate. For this reason, increasing our knowledge of liver regeneration mechanisms could bring significant benefits in the treatment of liver failure and may help patients needing large liver resections or transplantation.

In the present review, we propose an update on liver regeneration, cell-based regenerative medicine approaches, and bioengineering alternatives to liver transplantation, along with futuristic approaches to overcome hurdles in liver tissue engineering.

## 2. Liver Regeneration 

### 2.1. Overview of Liver Development

Hepatocytes and cholangiocytes, the two main liver cell types, are derived from the endoderm germ layer. This layer develops from the anterior primitive streak during gastrulation and is identifiable 6 h post-fertilization in zebrafish, by embryonic day 7.5 in mouse, and in the third week of human gestation [6]. The endodermal germ layer forms a primitive gut tube in which the regions of foregut, midgut, and hindgut can be identified. Fate mapping studies in mouse indicate that the embryonic liver originates from the ventral foregut endoderm by embryonic day 8.0 of gestation (e8.0) [6]. The hepatic endoderm cells, identified as hepatoblasts by e9.5, delaminate from the epithelium and invade the adjacent mesenchyme of the septum transversum to form the liver bud [7,8]. The hepatoblasts are bipotential cells and, during maturation, those residing next to the portal veins become biliary epithelial cells, while the majority of hepatoblasts in the parenchyma differentiate into hepatocytes [9]. During this process, the liver acquires its characteristic tissue architecture [10]. The balance in the numbers of hepatocytes and cholangiocytes from hepatoblasts is strictly controlled by integrated signaling and transcriptional pathways. The differentiation of hepatoblasts towards a biliary epithelial phenotype is controlled by the Jagged–Notch pathway [11,12], while hepatocyte differentiation is promoted by hepatocyte growth factor (HGF) and oncostatin M (OSM) [13]. Gradually, as the liver’s development proceeds towards the final stages of maturation, which begins by e13 and continues until several weeks after birth, there is a marked decline in the number of hepatoblasts [14]. However, some of the bipotent progenitor cells do not differentiate and gradually stop proliferating, establishing the pool of hepatic progenitor cells (HPCs) [15].

### 2.2. Homeostasis and First Line of Response to Injury 

The liver has a variety of functions fundamental to homeostasis, including bile secretion, metabolism, serum proteins production, glycogen storage, and drug detoxification. Since the Ancient Greek era with the famous “Prometheus” myth, the liver has been known to have a strong intrinsic regenerative ability in vivo. Thanks to a number of evolutionary protections, this physiological process of liver regeneration allows the recovery from even substantial hepatic damage caused by toxins or viral infections [16].

Hepatic regeneration, enabling the liver to continue to perform its complex functions despite a significant injury, is crucial to the survival of mammals and is therefore evolutionarily conserved and pathways leading to its completion are essentially redundant [17].

**Figure 1:**
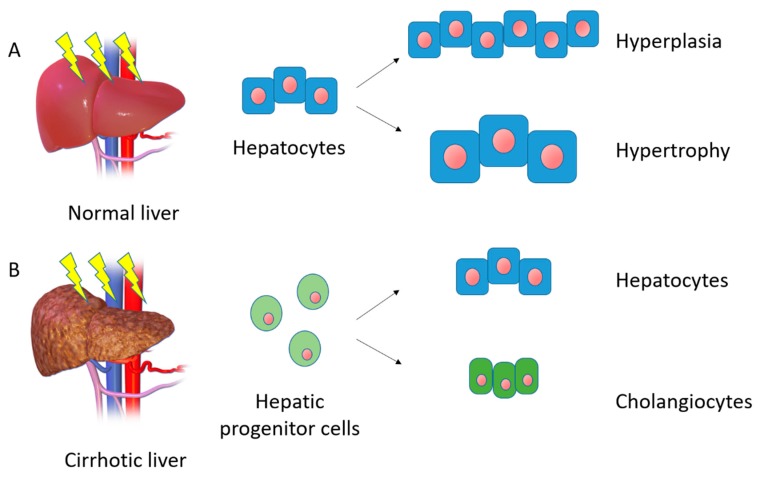
Schematic representation of mechanisms of liver regeneration. (**A**) After liver damage in normal conditions, the principal ways to restore hepatic mass are hyperplasia and hypertrophy. (**B**) In the cirrhotic liver, the normal regeneration process is impaired and hepatic progenitor cells are involved in restoring liver functions.

After the loss of tissue or an injury, the liver responds with fine-tuned pathways of regeneration via the activation of a wide array of signaling and transcriptional factors. As such, after surgical partial hepatectomy, the liver’s mass and function are restored within a week [16]. In epithelial tissues with a high turnover, such as the intestines and the skin, cellular renewal and tissue homeostasis is performed by a pool of stem cells. In the liver, however, the turnover is low with a mature hepatocyte having a life expectancy of about 200 days [18]. The general assumption, until recently, was that all mature hepatocytes were able to divide to ensure normal liver homeostasis [19,20]. Now the prevailing theory is that regeneration of the liver after resection is a compensatory hyperplasia rather than a true restoration of the liver’s original gross anatomy and architecture [21] (Figure 1A). The degree of hyperplasia is precisely controlled so that the process stops once an appropriate liver-to-bodyweight ratio has been achieved.

### 2.3. Hepatic Stem Cells and Second Line of Response to Liver Injury

Hepatic regeneration can be inhibited by several pathologic conditions. These include diabetes mellitus, malnutrition, aging, infection, chronic ethanol consumption, biliary obstruction and, more generally, chronic liver diseases. A common feature of all chronic liver diseases is progression to fibrosis, characterized by an increased production of matrix proteins, induced mainly by activated hepatic stellate cells, and a decreased matrix remodeling. With fibrosis there is usually diffuse inflammation and hepatocyte death, with evidence of an increase in the proportion of senescent hepatocytes, with the cell cycle arrested at G1/S transition. The rate of hepatocyte telomere shortening, which hampers cell division, has also been shown to correlate with the rate of progression of fibrosis [22]. Altogether, these data support the concept that the progression of liver fibrosis is associated with an impaired liver regeneration.

It has been hypothesized that, during chronic liver injury, when hepatocyte proliferation is impaired, the HPCs or oval cells orchestrate the regeneration process [23] (Figure 1B). The existence and regenerative potential of HPCs have been questioned, however. Farber was the first to report on the presence of a liver progenitor cell population in 1956, when he identified small cells with a high nucleus-to-cytoplasm ratio in the liver, that he called “oval cells” [24]. Subsequent works [25,26] demonstrated that these cells were activated in animal models of liver injury and had a bipotential ability to differentiate into hepatocytes and bile duct cells. Most of the data have come from animal models with the chemically-induced inhibition of native hepatocytes, in conjunction with the stimulation of liver regeneration. Lineage-tracing experiments have localized the adult human equivalent of these progenitor cells in the canal of Hering in the periportal regions of the hepatic lobules [27]. 

HPCs have the capacity to differentiate into hepatocytes and biliary cells in vitro, and to form hepatocyte buds, repopulating the damaged parenchyma in specific situations in vivo, in what is called “oval cell proliferation" in rodent models, and a “ductular reaction" in humans [28,29,30].

On activation, when the adult hepatocytes are unable to regenerate the injured liver, due either to senescence or cell cycle arrest, the HPCs proliferate in the portal zone and migrate towards the central vein in the liver lobules, gradually going through different states of maturity and function along the way, according to the so-called “streaming liver hypothesis” [29,30,31].

While the above is the most widely-accepted theory, work by Kuwahara et al. [32] suggests that it may be an oversimplification and that the liver might have a multi-tiered system of regeneration. There may be up to four potential stem cell niches in the canal of Hering, the intralobular bile ducts, the periductal mononuclear cells, and the peribiliary hepatocytes.

However, despite the accumulating evidence of HPC proliferation in liver injury, the extent of these cells’ contribution to the natural history of human liver disease, and the triggers that activate this cell population are still not well understood.

### 2.4. Liver Regeneration, Inflammation, and Gender

Liver regeneration is closely linked to inflammation. Indeed, hepatic inflammation is a complex process originating in response to specific stress stimuli, which modulates the outcome of liver damage [33]. The inflammatory response can have both hepato-protective and detrimental effects. A controlled inflammatory reaction could be adjuvant to tissue regeneration, promoting the re-establishment of homeostasis. On the other hand, excessive and permanent inflammation could exacerbate the severity of hepatic parenchymal damage contributing to the irreversible decline of liver function [34]. Given its fundamental role, the inflammatory process is strictly controlled at a molecular and cellular level. Both resident (Kupffer) cells and circulating immune cells (lymphocytes and monocytes) are involved [33]. Among the molecular pathways involved in liver regeneration, IL-6 and IL-22 produced by activated natural killer (NK) and T cells in the liver induce activation of the signal transducer and activator of transcription 3 (STAT3) [35,36], while interferon-γ (IFN-γ) produced by B and T cells activates STAT1, inhibiting liver fibrosis and regeneration [37,38].

In a recent study [39], we demonstrated that a different immune response (in terms of the composition and maturation status of the cells involved) influence liver regeneration in males and females. The liver is known to be a gender-dimorphic organ in mammals, exhibiting sex-related differences in various aspects, such as the profile of steroid and drug metabolism [40], the number of hepatocytes and Kupffer cells [41], and the regeneration rate [42,43]. We demonstrated that female mice showed a more rapid recruitment of monocytes and F4/80^high^CD11b^high^ cells, and that the delay in recruitment of the same cells in male mice was controlled directly by the androgen receptor. Evidence from patients with drug-induced liver injury (DILI) confirms these observations, suggesting that males show a delay in regenerative response to an acute liver injury, possibly related to a maturation shift in monocytes. These findings might provide interesting starting points for new, gender-specific biomarkers, or for novel therapeutic interventions targeting monocyte recruitment or sex-hormone signaling [44,45]. Larger observational or prospective trials are needed, however, to better understand sex-dependent immune mechanisms in DILI.

## 3. Alternatives to Liver Transplantation 

Liver transplantation (LT) is a widely-recognized treatment for patients with end-stage liver disease. Since the first success story reported by Thomas Starzl in 1967 [46], the short- and long-term outcomes of transplanted patients have gradually improved thanks to advances in the management of immunosuppressant therapies, more appropriate donor-recipient matching, and a better treatment of post-transplant comorbidities [47].

In recent years, we have witnessed an increasing number of patients worldwide on the waiting list for a transplant, but the number of available donors has not increased accordingly [48]. This gap between the patients needing a transplant and the donor organs available is a key issue in LT, with a mortality risk while on the waiting list of approximately 15% [49,50]. 

**Figure 2:**
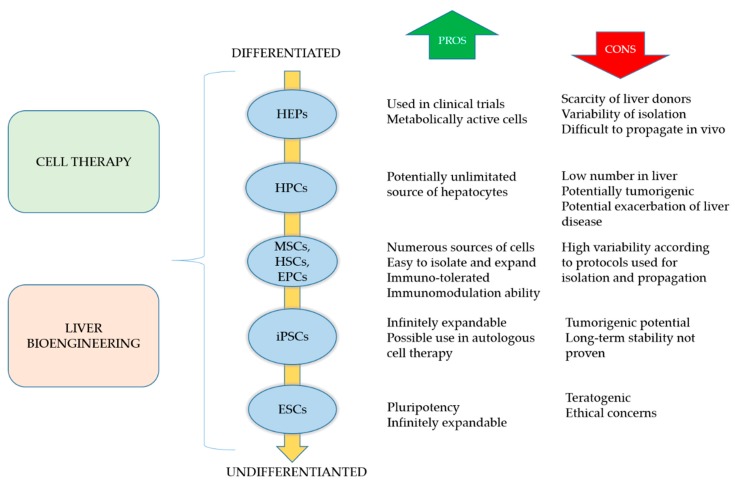
Schematic representation of the principal cell sources available for cell therapy and liver bioengineering, with brief description of their pros and cons. HEPs: hepatocytes; HPCs: hepatic progenitor cells; MSCs: mesenchymal stem cells; HSCs: hematopoietic stem cells; EPCs: endothelial progenitors cells; iPSCs: induced pluripotent stem cells; ESCs: embryonic stem cells.

Liver regenerative medicine could cope with the donor shortage by using innovative approaches based on cell therapy and tissue/organ engineering. In the following sections, we briefly describe these ground-breaking alternatives to LT. Figure 2 summarizes the principal cell sources available for cell therapy and liver bioengineering, with their pros and cons. 

### 3.1. Cell-Based Regeneration Therapy

As the demand for donor organs grows, therapeutic alternatives to liver transplantation must be sought. One such possible alternative is cell therapy, which may have two roles in the treatment of chronic liver diseases. Its first role is to control disease progression by stimulating endogenous regeneration and inhibiting fibrosis, thus ideally eliminating the need for liver transplantation [51]. When liver transplantation cannot be avoided, cell therapy may act as a bridge to surgery supporting liver function and, potentially, reducing the waitlist mortality rate. During the last ten years, hepatocytes, macrophages and stem cells have been transplanted with vary-ing degrees of success. 

#### 3.1.1. Hepatocytes

Primary hepatocytes are the cells traditionally used for cell therapy in chronic liver diseases. It has been demonstrated that splenic or portal vein infusions of hepatocytes could induce modest reductions in ammonia levels and encephalopathy in both animal models and humans [52]. However, there are several important limitations to the use of human hepatocytes in the treatment of chronic liver diseases. One of the most important drawbacks is the difficulty of isolating a sufficient quantity of high-quality, metabolically-active cells. Hepatocytes are typically harvested from livers not suitable for transplantation, with a consequent variability in their quantity and quality [53]. Hepatocytes also rapidly lose their proliferative ability when cultured in vitro, and they are sensitive to freeze-thaw damage so their viability and engraftment are affected by culture and cryopreservation methods [54]. Innovative technologies that can expand, maintain, mature, and create hepatocytes in vitro, or alternative sources of cells are consequently required for future cell-based therapies for liver diseases.

#### 3.1.2. Macrophages

Evidence has emerged from numerous human and animal studies of liver fibrosis being a two-way process and potentially reversible. The main regulator of this dynamic fibrogenesis-fibrosis resolution paradigm seems to be the hepatic macrophage [55,56]. This apparently dichotomous effect of macrophages in liver fibrosis is attributable to the balance of profibrotic and restorative macrophages [57]. A better understanding of the mechanisms controlling this process could yield novel monocyte/macrophage-based cell therapies.

Technological advances in the stem-cell field could lead to therapeutic approaches based on the autologous propagation of monocytic populations, or possibly their derivation from embryonic stem cell components. A monocyte/macrophage-based approach to damping liver fibrosis has already been attempted in animal models. Thomas et al. [58] examined the therapeutic potential of exogenous bone marrow (BM) cells, and those of the monocyte-macrophage lineage in particular, in a mice model of chronic liver injury. They found that the intraportal administration of differentiated BM-derived macrophages (BMMs) improved liver fibrosis, regeneration, and function via a wide range of reparative pathways, with a therapeutic benefit. On the other hand, liver fibrosis was not significantly ameliorated by the infusion of macrophage precursors, and it was even exacerbated by whole BM cells. Thanks to paracrine signaling from the BMMs to larger populations of endogenous cells, their effect was amplified. As a consequence, a modest number of donor BMMs could exert whole-organ changes—encouraging a translational perspective and suggesting a future clinical potential.

#### 3.1.3. Pluripotent Stem Cells 

Embryonic stem cells (ESCs) are pluripotent cells derived from the inner cell mass of the blastocyst. These cell were first characterized in 1998 by Thomson et al. [59]. They have pluripotency and can potentially differentiate into all somatic cells [60]. Numerous studies have demonstrated the differentiation of ESCs into hepatocyte-like cells that express a number of hepatocyte-related genes and mimic liver function [61,62,63,64,65]. ESC-derived hepatocytes also have the typical morphology of mature hepatocytes and are able to colonize liver tissue after transplantation, promoting the injured liver’s recovery via cell replacement and stimulating endogenous regeneration [63,66,67,68]. Despite these promising results and the favorable characteristics of human ESCs, such as a good resistance to cryopreservation, practical and ethical barriers have always precluded their application in clinical practice. 

Human induced pluripotent stem cells (iPSCs) have recently emerged as a way of bypassing the ethical concerns associated with the use of ESCs [69]. The iPSCs are derived by reprogramming mature somatic cells induced by different transcription factors [70]. Their characteristics of self-renewal and pluripotency make iPSCs good substitutes for ESCs, and an appealing source of normal human cells that can differentiate into virtually any somatic cell type, including hepatocytes. The hepatocyte-like cells (HLCs) derived from human iPSCs could provide a stable source of hepatocytes for multiple applications, including cell therapy, disease modelling, and drug safety screening [71,72]. Protocols adopted to differentiate human ESCs and human iPSCs into HLCs mimic the developmental pathway of the liver during embryogenesis, and have vastly improved in recent years. Nevertheless, several issues regarding the safety and reproducibility of iPSCs still need to be settled before their real clinical application, including tumorigenicity and teratoma formation, the debate on their immunogenicity, long-term safety and efficacy, and the optimal reprogramming and manufacturing processes [73,74,75]. Constant progress is nonetheless being made in reprogramming technologies, and in new and improved manufacturing methods. 

#### 3.1.4. Adult Stem Cells

Stem cells are valid alternative sources of cells for the treatment of liver diseases. They could potentially be involved in modulating the liver’s regenerative processes to reduce scarring in cirrhosis, and to down-regulate immune-mediated liver damage. Stem cells could also be differentiated into hepatocytes for cell transplantation, or used in extracorporeal bioartificial liver systems [76].

Different types of adult stem cells have been tested over the years, including hematopoietic stem cells (HSCs), mesenchymal stem cells (MSCs), endothelial progenitor cells (EPCs), and hepatic progenitor cells (HPCs) [77,78,79,80]. 

HSCs are the predominant population of stem cells in bone marrow, and express the surface marker CD34. HSCs can easily be isolated in the bloodstream after treatment with mobilizing agents, the most widely-studied and often-used of which is the granulocyte-colony stimulating factor [81]. Hepatocyte-like cells derived from HSCs have been demonstrated to support liver regeneration [82,83]. Different mechanisms have been suggested, such as the de novo generation of hepatocytes through transdifferentiation or the genetic reprogramming of resident hepatocytes through cell fusion [84,85]. However, the most plausible hypothesis is that the clinical benefit of HSC therapy occurs through paracrine signaling interactions involving various cytokines and growth factors, that stimulate regeneration and neoangiogenesis [86,87]. 

Endothelial progenitor cells (EPCs) can be found in both peripheral blood vessels and bone marrow, and their main function is to participate in the neovascularization of damaged tissue [88,89]. In the context of cell therapy for liver diseases, one animal study demonstrated that the transplantation of EPCs led to a lessening of liver fibrosis [79]. ESCs are also able to promote hepatocyte proliferation and increase matrix metalloproteinase activity [90]. All these effects are related to an increased secretion of specific growth factors [91,92].

Another promising cell treatment for liver diseases is based on mesenchymal stem cells (MSCs), a population of multipotent progenitors capable of differentiating towards adipogenic, osteogenic and hepatogenic lineages, with a low immunogenicity [93]. Bone marrow is considered the main source of MSCs [94], but alternative sources are being examined, such as adipose tissue [95], placenta, amniotic fluid, umbilical cord blood, and umbilical cord [96,97]. Our research has focused on umbilical cord MSCs. We demonstrated in an animal model that, when systematically administered, these cells can repair acute liver injury [97]. The ability of the same cells to repair tissue damage was also demonstrated in a chemically-induced intestinal injury in immunodeficient mice [98]. We and other authors have demonstrated that MSCs have the capacity to provide both metabolic and trophic support due to their potential for hepatocytic differentiation, and their secretion of anti-inflammatory, anti-apoptotic, immunomodulatory, and pro-proliferative factors [97,98,99]. This leads to liver function being restored via the repair of damaged tissue, the suppression of inflammation, and the stimulation of endogenous regeneration through paracrine effects [100].

Cell-based therapy using HPCs could potentially regenerate the liver during chronic diseases. Multiple protocols have been established for isolating HPCs in fetal and rodent models, and cell differentiation protocols are available for progenitor cells derived from the human liver or biliary tree [101,102,103]. Due to the low number of these cells in the liver, the use of autologous HPCs is probably unfeasible. The use of expanded fetal or syngeneic HPCs is more likely, though this approach raises questions regarding the engraftment rate of transplanted cells, and the need for immunosuppressant therapy. Despite the theoretical feasibility of such approaches, we still have only a limited understanding of HPCs, their precise role in liver pathophysiology, and how the entire process of regeneration/differentiation is regulated. Given the possible disadvantages of HPC activation, which might exacerbate disease progression or prompt the onset of cancer [104], all these issues warrant further study and careful examination before any therapeutic approaches could be applicable.

#### 3.1.5. Hepatic Organoids 

Considered as a bridge between liver cell therapy and liver bioengineering, hepatic organoids are functional three-dimensional (3D) in vitro models of the liver consisting of a spherical monolayer of epithelium that preserves the key physiological features of the liver [105]. Liver organoids are typically obtained by isolating and expanding stem cells or hepatic progenitor cells. 

Liver organoids show a limited spontaneous differentiation during maintenance and expansion. For this reason, protocols for establishing organoids were divided into two steps. The first relied on proliferation culture conditions for the establishment and expansion of hepatic organoids. Then, in a second step, proliferative signals were removed, and differentiation towards hepatocyte-like cells was induced. These culture conditions enabled organoids to be obtained with 30–50% fulfilling hepatic characteristics [106], but without the complete functional repertoire of adult hepatocytes—a drawback shared by HPC-to-hepatocyte differentiation. 

Differentiated hepatic organoids transplanted into mouse models of liver failure have demonstrated a capacity for engraftment and repopulation of the damaged liver, with partial rescue of liver function [105]. Equivalent human liver organoids transplanted into mice with acute liver damage were able to produce human albumin and alpha-1-antitrypin, with secretion levels comparable with those after the transplantation of adult hepatocytes [101].

Three-dimensional liver tissue has also been engineered by using human iPSCs to derive hepatocytes in co-culture with mesenchymal and endothelial cells [107]. When transplanted into mice, these liver buds were vascularized and matured to synthesize serum proteins and carry out detoxifying functions. 

Current research is aiming for the clinical application of liver buds suitable for hepatic administration via the portal vein in patients in need of a liver transplant [108]. Among the different cell sources, adult stem cells directly derived from hepatic tissue are preferred. Indeed, drawbacks of human iPSCs or trans-differentiated cells used in the design of clinical solutions concern their exposure to genetic modifications through reprogramming factors, and their genomic instability, particularly in long-term cultures [109].

Moreover, liver organoids provide a novel platform for research on: 1) liver development and regeneration; 2) detoxification and metabolism; 3) liver disease modelling; and 4) adult stem cell biology.

### 3.2. Liver Tissue Bioengineering 

Tissue engineering could offer various solutions for reducing the waiting list by creating biocompatible scaffolds and extracorporeal liver devices suitable for either in vitro or in vivo applications [110]. 

In the last two decades, a growing number of studies demonstrated that 3D cultures have a number of advantages over traditional two-dimensional (2D) cell cultures [110,111]. A physiologically 3D microenvironment is crucial to the development of in vitro tissue models, particularly for such complex tissues as the liver, in which the interaction between hepatocytes, hepatic stellate cells, and extracellular matrix (ECM) creates the microenvironment of the hepatic lobules [112].

The search for efficient biocompatible scaffolds aims to create organic or polymeric constructs that mimic the liver ECM and replicate functional characteristics such as cell adhesion, viability, growth, and proliferation. The principal strategies are based on biomaterials such as polymer-based 3D constructs, decellularized ECM, or bioprinting 3D constructs. 

Another recent approach involves the development of bioreactors to improve various functions of hepatocytes that are seeded in constructs. In bioreactors, a real 3D microenvironment niche is created to improve cell attachment, growth, and proliferation, with a marked improvement in liver metabolism and function [110]. A more sophisticated technology is the liver-on-chip: A combination of bio-reactor techniques and microfluidic devices to sustain the phenotype of hepatocytes and liver-specific functions in long-term culture [113]. 

**Figure 3:**
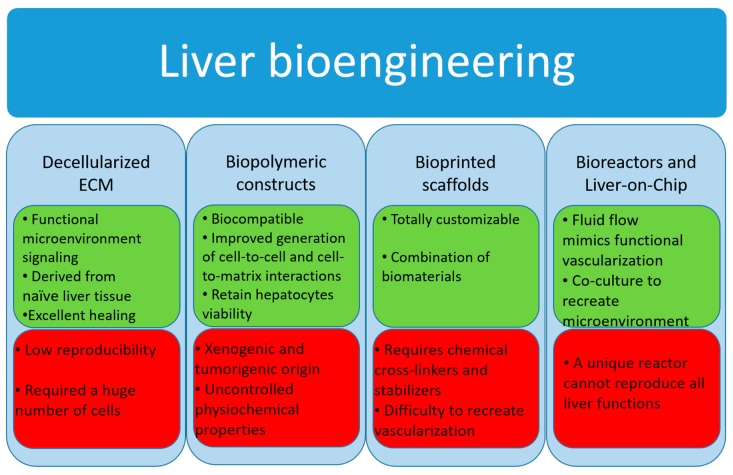
Main pros (green boxes) and cons (red boxes) of the principal liver bioengineering approaches. ECM: extracellular matrix.

Below we provide an overview of such bioengineering approaches, and Figure 3 shows the main pros and cons of each of them.

#### 3.2.1. Decellularized Extracellular Matrix 

A new approach to liver regenerative medicine involves generating 3D organs with a decellularized, native liver bioscaffold that can be repopulated with parenchymal and non-parenchymal cells [114]. The liver’s native ECM has a complex composition and topography, serving as a structure for cell-ECM adhesion, interaction, and polarity, with implications for the regulation of cell morphology, proliferation, differentiation, and viability interactions [115]. Donor organs unsuitable for transplantation are used to create whole-liver scaffolds which are subsequently reseeded with healthy cells to create transplantable liver grafts. The scaffolds maintain the native liver architecture and ECM composition, which allows for proper cell homing and function. Decellularization techniques were introduced in the 1980s [116], but the concept of whole-organ decellularization was developed later by Ott and colleagues in mice hearts [117]. This technique was later adapted for liver engineering purposes [118], with the preservation of the chemical composition and structure of the ECM with structurally intact vessels, and bile ducts. This bioscaffold was then recellularized with hepatocytes and endothelial cells. The recellularized graft transplanted in vivo and perfused ex vivo demonstrated mature liver functions. Further improvements in the technique were obtained over the years, such us multistep cell seeding, the use of stem cells (MSCs, fetal hepatocytes, iPSCs) [119,120,121], optimization of the decellularization cocktail, and perfusion without any thrombus formation [122]. The feasibility of this technique was also demonstrated in larger animal models [123], and even in humans [124], bringing the approach to clinical scale. 

All these studies demonstrated that decellularized livers hold great potential as a therapeutic approach, but numerous pitfalls remain. First, the technique allows for the successful seeding and culture of hepatocytes, but colonization of the bile duct with functional cells and the achievement of an intact vascular network remain to be perfected. Another important issue before whole liver bioscaffolds can be used in clinical practice is the lack of a suitable source of cells, which should be readily available and renewable because successful liver recellularization demands hundreds of millions of cells. The limited availability and inability to expand primary hepatocytes has led researchers in the field to search for a new cell source. Although many groups have attempted to overcome the problem by using fetal liver cells, stem cells or iPSCs, the production of such huge numbers of hepatocytes is still far beyond current technical capability. 

Another hurdle that should be promptly addressed is “sample to sample” variation due to the unique condition of each donor deriving from the use of discarded livers [125]. The next goals of bioengineering research will be to solve these problems. 

#### 3.2.2. Biopolymer Constructs 

In modern tissue engineering, efforts are being made to make natural biomaterials mimic the natural hepatic ECM. The main components of these scaffolds are collagen and hyaluronic acid. The latter strongly supports cell attachment, proliferation, differentiation, growth, and migration. Immature and mature hepatocytes express CD44, the surface receptors for hyaluronic acid, so biopolymers with hyaluronic acid and its derivatives have more adhesive power for hepatocytes. They can retain hepatocyte viability for 4 weeks [126].

Other natural biomaterials used in the construction of bioactive scaffolds are alginate, chitin, chitosan, silk, Matrigel^®^, and sponge. Matrigel^®^ is a scaffold consisting of a mixture of ECM proteins derived from the basal membranes of murine chondrosarcoma, which contains laminin, heparan sulfate proteoglycan, and collagen type IV [127]. It has been used in numerous studies to culture hepatocytes and induce the hepatic differentiation of stem cells [97,128]. 

Although hydrogels formed by natural biomaterials such as alginate and Matrigel^®^ are biocompatible and improve the generation of cell-to-cell and cell-to-matrix interactions, they have some important limits that prevent their clinical application. The main shortcomings of such biomaterials are their uncontrollable physicochemical properties, degradability, lack of regenerative ability, and inconsistent mechanical properties. Moreover, due to the xenogenic and tumorigenic origin of Matrigels, they are not an optimal support for clinical applications in liver bioengineering [129]. 

By comparison with natural biomaterials, synthetic materials offer a wide range of properties and a better control over them. Scaffolds containing biodegradable polymers, such as polylactic acid, polyglycolic acid, polyanhydrides, polyfumarates, polyorthoesters, polycaprolactones, poly-L-lactic acid, and polycarbonates facilitate cell regeneration, transplantation, and degradation on time [130]. The biocompatibility of bioengineered matrices and scaffold adhesion properties could also be improved by chemically modifying these polymers (e.g., by incorporating proteins and special bioactive domains), stimulating cell attachment and migration, and thereby facilitating liver tissue repair [131]. 

While natural and synthetic materials support the successful culture of hepatocytes, these constructs fail to perfectly reproduce the microenvironment of the liver essential to a functional liver cell activity. For this reason, their therapeutic potential is limited. 

#### 3.2.3. Bioprinted Scaffolds

Although the use of biomaterials in 3D culture has improved the settings for liver tissue engineering, it has some limitations. These include the difficulty of creating complex biological structures and designs due to size, material, compositional, and technological constraints [132]. An innovative solution to these problems involves using bioprinted scaffolds, tissue-mimicking constructs created by means of a bioprinting process with biocompatible materials (i.e., bio-inks) [133]. Advances in bioprinting technology have enabled the creation of more complex 3D structures using combinations of different biomaterials and cell types [134]. The chance to totally customize the prints also guarantees the complete personalization of such scaffolds and their applications. The available bioprinting modalities include extrusion, inkjet, and laser-assisted bioprinting [135]. Extrusion bioprinting, the most often-used bioprinting modality in biomedical research, allows for a strong degree of customization with few restrictions on the cells used [134]. The choice of biomaterials is more restrictive, however, as they are either easy to print or ideal for cell culture, but typically not both [136]. The ideal characteristics of bio-inks for extrusion bioprinting are viscosity to enable printing, associated with an adequate elasticity to maintain their structure, while also maintaining cell viability and supporting cell function [132].

The most common biomaterials used for bioprinting are collagen, alginate, polyethylene glycol (PEG), hyaluronic acid, fibrin, gelatin, or polycaprolactone, each with unique properties [133]. With the exception of collagen, these biomaterials need the addition of a cross-linker that could adversely affect the cells. For this reason, they should be appropriately balanced to guarantee the best biocompatibility of the bio-ink being used [133]. Although collagen is an ideal material for in-vivo-like tissue replication, it is a poor bio-ink because it has a time- and temperature-sensitive cross-linking [137]. A multi-component hybrid bio-ink is therefore a potential solution for achieving ideal physiological relevance and bio-printability. Unfortunately, durable 3D construct fabrication requires the incorporation of chemical stabilizers, such as polycaprolactone, showing the limitations of bio-inking technologies in mimicking both the biochemical composition and the complex 3D structure of the liver. 

Another important challenge in 3D bioprinting is how to fabricate and mimic cellular microenvironments from molecular to macroscopic scales for tissue engineering and regenerative medicine. Using this approach, the researcher aims to create a whole functional liver suitable for transplantation, but some important issues, such as vascularization, should be addressed before this methodology can really be implemented. 

### 3.3. Bioreactor Systems 

Despite the great progress made in biomaterial development for tissue engineering, some challenges need to be overcome. The most important limiting parameter in tissue engineering and bioprinting concerns vascularization [138]. Without suitable vascularization, cells are subject to hypoxia, toxemia, apoptosis, and immediate cell death. The bioreactor approach aims to overcome this limitation. In fact, the bioreactor involves a designed or programmed fluid flow as an integral part of the culture format. The flow in perfusion bioreactors enables a continuous exchange of nutrients, a better oxygen delivery, and a physiological shear stress, influencing cell function in ways that are impossible to achieve in static culture formats [139].

The evolution of bioreactor technologies has paralleled advances in the development of functional biomaterial scaffolds [140]. The scaffold not only provides an adhesion surface for cells, but also profoundly influences cell shape and gene expression relevant to cell growth and liver-specific functions. Moreover, when placed as a separation between cells and the medium, the scaffolds act as a modulator for water and nutrient transport from the medium to the cells, and discharge waste metabolites from the cells to the medium [141].

Four principal types of bioreactors have been used for liver cell culture: 1) flat plate and monolayer; 2) hollow fiber; 3) perfused beds and scaffolds; and 4) encapsulation and suspension. With the exception of type 1, the other bioreactors enable the 3D monoculture or co-culture of hepatocytes under tissue-specific mechanical forces (pressure, shear stress, flow) [142,143]. Some of these bioreactors have been used as bioartificial livers, charged with various types of liver cells, as a bridge for patients with acute liver failure awaiting transplantation [144]. Now the challenge is to use cell-based bioreactors as in vitro screening systems for drug toxicity, metabolism evaluation and potential clinical treatments.

Some parameters are crucial to hepatocyte vitality and functionality, including various biophysical factors such as oxygenation, hemodynamics, and shear stress. Perfusion in bioreactor devices enables the establishment of oxygen gradients and hepatic zonation, resulting in graded CYP expression and metabolism [145,146]. A controlled oxygen gradient from 25 to 70 mmHg inside a hepatic bioreactor creates a functional hepatocyte zonation similar to what is observed in vivo. Cell oxygenation could be partially controlled by varying the medium flow rate, but may consequently exert a shear stress on the hepatocytes. Flow rate should be carefully controlled since cell damage can occur. Hydrodynamic stress induces ECM remodeling, scaffold degradation and changes in tissue composition, influencing the device’s structural and mechanical properties. On the other hand, low flow rates limit the oxygen supply, lead to nutrient deficiency, and reduce cell viability and survival probability [146]. 

The co-culture of hepatocytes and non-parenchymal cells is important for the reorganization of hepatocytes in culture by secreting cytokines, nitric oxide, and matrix components [145,147]. Co-culture is also useful for inducing liver-specific functions, preserving maximal levels of functional adhesion molecule expression, and reducing the number of cells needed for a bioartificial liver [144]. 

The main limitation of the bioreactors is that not all critical liver functions can be replicated on the desired level as yet. For this reason, based on the present state of the art, a unique bioreactor that can faithfully reproduce all liver functions is still lacking.

### 3.4. Micro-Bioreactors and Liver-on-Chip 

The combination of nanotechnology, microchips, and microfluidics in a single device has great potential for applications in liver tissue engineering. Various strategies have been developed to obtain micro-bioreactors. Microsystems technology has been used to fabricate 2D or 3D culture devices by using different types of materials, like silicon, silicone elastomer, and biocompatible and biodegradable polymers. Such systems typically exhibit laminar flow, similar to the environment in vivo, and allow the creation of microfluidic channels with larger surface-to-volume ratios suitable for oxygen and nutrition supply [113].

Other interesting systems that exploit microfluidic technology are the so-called “liver-on-chip” devices [148]. These systems consist of microchambers containing engineered tissue and living cell cultures interconnected by a microfluidic network. Such organs on chips enable the study of human phys-iology in an organ-specific context, and the development of novel in vitro disease models. They have the potential to serve as replacements for animals used in drug development, toxin testing, and screening for biothreats and chemical warfare agents [149]. 

## 4. Conclusions

In conclusion, regenerative medicine and bioengineering are cutting-edge technologies that look promising as a final solution to the treatment of end-stage liver diseases. A better understanding of liver regeneration and the development of in vitro systems that successfully mimic hepatocyte expansion and differentiation will make autologous cell therapy a feasible alternative to liver transplantation. The current scenario is also moving towards the successful development of whole bioengineered livers and their effective use in clinical practice in lieu of liver transplantation.
